# Effect of sonic hedgehog/β-TCP composites on bone healing within the critical-sized rat femoral defect

**DOI:** 10.3892/etm.2013.923

**Published:** 2013-01-23

**Authors:** JÖRG WARZECHA, CAROLINE SEEBACH, ARMIN FLINSPACH, FLORIAN WENGER, DIRK HENRICH, INGO MARZI

**Affiliations:** Department of Trauma Surgery, Johann Wolfgang Goethe University, D-60590 Frankfurt am Main, Germany

**Keywords:** bone substitute material, tricalcium phosphate, sonic hedgehog, bone morphogenetic protein-2, rat femur defect, three point bending test

## Abstract

The creation of entirely synthetically derived bone substitute materials which are as effective as autologous bone grafts is desirable. Osteogenesis involves the concerted action of several proteins within a signaling cascade. Hedgehog proteins act upstream of this cascade, inducing the expression of various bone morphogenetic proteins (BMPs) and promoting physiological bone healing. Therefore, the hypothesis that hedgehog signaling in bone defects improves bone healing more than BMP signaling alone was tested. Recombinant N-terminal sonic hedgehog protein (N-SHh), BMP-2 or a combination of the two was added to β-tricalcium phosphate (β-TCP) and 5-mm femoral midshaft defects in nude rats were filled with these composites. The defects were stabilized with mini-plates. After eight weeks, the animals were sacrificed and the femora were explanted. The radiological evaluation was followed by a three-point bending test and histological examination. BMP-2/β-TCP composites showed a trend of increased stiffness compared with the controls (β-TCP without protein). N-SHh/β-TCP composites had lower stiffness compared with the control group and the N-SHh/BMP-2/β-TCP composites also had lower average stiffness compared with the controls (all not significant). Histomorphometry, however, revealed abundant cartilage and bone core formation in the N-SHh-composite groups. The sum of the new cartilage and bone was highest in the combination group N-SHh/BMP-2 (not significant). The addition of N-SHh to bone substitute materials appears to delay bone healing at the applied concentration and observation time but also showed a trend for higher amounts of ossifying cartilage.

## Introduction

Hedgehog proteins are crucial in the embryonic development of animals, from insects to mammals (1). Furthermore, hedgehog proteins are postnatally involved in physiological bone growth as well as in fracture healing (2–4). *In vitro*, sonic hedgehog protein (SHh) causes the proliferation and differentiation of mesenchymal stem cells into the osteoblastic lineage by upregulating bone morphogenetic proteins (BMPs) via SMAD signaling (5). Previous studies have shown that the combination of recombinant N-terminal SHh (N-SHh) with BMP-2 synergistically induces the expression of osteogenic markers in progenitor cells, whereas the transplantation of N-SHh into the muscles of mice failed to induce ectopic bone formation after two weeks (6). In turn, the implantation of dermal fibroblasts expressing hedgehog proteins into nude mice induced ectopic bone formation (7). The mechanism for hedgehog proteins in bone formation *in vivo* remains unclear.

The expression of SHh by transduced fibroblasts appears to be a reliable method for inducing ectopic bone formation, since a further study demonstrated the closure of calvarial bone defects in rabbits using SHh-expressing fibroblasts (8). However, in clinical practice, the use of genetically modified cells does not yet appear to be adequate. Instead, the application of osteoinductive proteins to porous synthetic bone substitute materials is a promising approach.

Based on past experience, a combination of BMPs with β-tricalcium phosphate (β-TCP), a bone substitute material, works synergistically to yield new bone in animals (9) and has good release properties, as well as inducing higher amounts of bone formation than are obtained with a combination of BMP-2 and hydroxyapatite (10).

The application of BMP-2 to bone defects in humans is already used in clinical practice. However, the benefit is not clear and adverse reactions, including infection, pain, heterotopic bone formation and immunogenic reactions, may be observed (11). One explanation for this limitation is that BMP-2 as a monotherapy requires higher concentrations to work than are required in physiological bone healing.

We hypothesized that the application of commercially available N-SHh protein (alone or in combination with BMP-2) to β-TCP would induce osteogenesis in bone defects in a superior manner to a combination of β-TCP and BMP-2.

## Materials and methods

### Scaffold preparation

#### Scaffold

β-TCP is one of the most commonly used materials for bone tissue engineering and it has osteoconductive properties (12). β-TCP is completely biodegraded over a period of up to two years, depending on the host’s metabolism (13). β-TCP bodies for small animal studies (chronOS; granule size, 0.7–1.4 mm) were obtained from Synthes (Dübendorf, Switzerland). The bodies had a porosity of ∼60%. The macropores had a diameter of ∼100–500 *μ*m and the micropores were <5 *μ*m. The weight of 0.5 cc chronOS (0.7–1.4 mm) was ∼0.5 g. Mesenchymal stem cells adhered favourably to chronOS *in vitro* (14).

#### BMP-2

Recombinant human BMP was purchased from R&D Systems (Wiesbaden, Germany). The human protein was used since BMPs are highly conserved across animal species. In particular, mature human, mouse and rat BMP-2 are 100% identical at the amino acid sequence level.

#### SHh

Recombinant human N-SHh was also obtained from R&D Systems. SHh proteins are highly conserved at the N-terminus across vertebrates (99%) and studies support that they are also conserved in their functional properties (15).

#### Composite preparation

The BMP-2 and N-SHh were dissolved in phosphate-buffered saline, yielding a stock solution comprising 20 *μ*g/ml BMP-2 and 50 *μ*g/ml N-SHh. According to the literature, these concentrations are only marginally effective and thus were suited to examining whether a synergistic effect was observed.

Next, the β-TCP was soaked with this solution. The soaked β-TCP was dried under a hood overnight, then packaged in 50 *μ*g (i.e. 2.5 *μ*g N-SHh and/or 1 *μ*g BMP-2) portions. These portions were stored at −20°C until use. Previous studies have shown that these preparations retain their osteoinductive activity (9,10).

### Surgical procedure

#### Animals

For the critical-sized defect model athymic nude rats (RH-Foxn1^rnu^) were used so that immunological reactions to the human N-SHh were entirely excluded. The animals were purchased from Harlan Laboratories (Harlan Winkelmann GmbH, Kreuzelweg, NM Horst, The Netherlands) This animal model is T-cell deficient but has normal B-cell function.

The animals were divided into four groups (Table I). The first group served as a control and the critical-sized femoral defects in this group were filled only with inorganic bone substitute material (β-TCP). The second group was a test group. The femoral defects of these animals were filled with β-TCP combined with N-SHh. The third group also served as a test group and the femoral defects were filled with β-TCP impregnated with N-SHh and BMP-2. In the fourth group, the defects were filled with β-TCP and BMP-2.

#### Surgery

**Figure 1:**
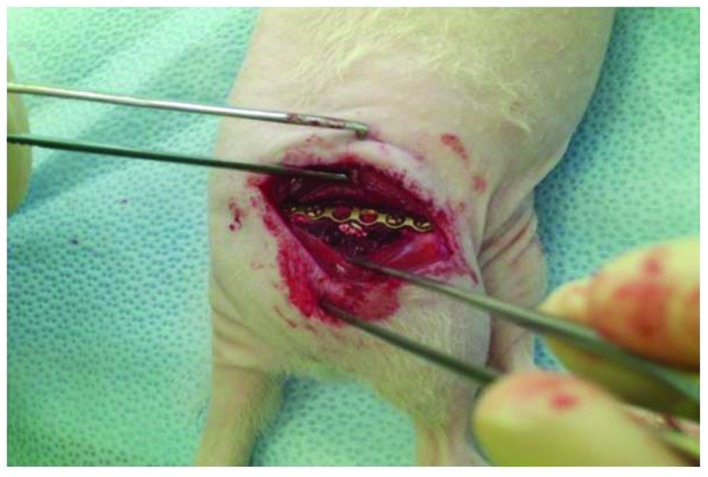
Intraoperative site of the critical-sized defect, stabilized with a mini-plate and filled with bone substitute material.

Under general anesthesia (ketamine/xylazine i.p.) and aseptic conditions, the anterolateral aspect of the right rat femur was dissected. A plate was then positioned on the femur and fixed with four screws, leaving two holes in the midshaft free. Next, the screws and plate were loosened and two 5-mm osteotomies of the mid shaft femur were performed with a liston-key. A 5-mm empty segmental defect in a rat femur does not heal (defined as a critical defect) as demonstrated in previous studies (16). The plate was then readapted to the femur and the screws were tightened. Protein-impregnated scaffolds were implanted into the segmental defects (50 *μ*g each) and the wound was closed with absorbable suture material (Fig. 1). The animals received metamizole in their drinking water as pain treatment over the subsequent days. The rats were housed for eight weeks under standard conditions and nutrition.

#### Osteosynthesis materials

The plates and screws were obtained from Synthes (LCP compact hand). The smallest titanium plate (1.0) with twelve non-locking holes was used. The plate was cut so that a six-hole-plate was used for each femur. The screws were self-cutting and had a diameter of 1.3 mm and a length of 6 mm. The decision was made to stabilize the critical-sized defect with a plate rather than an external fixator since plates appeared to be more comfortable for the rats and also exhibited greater stability (17).

### Evaluation

The animals were sacrificed after eight weeks by inhalation anesthesia and following i.p. pentobarbital injection. The femora were explanted and freed from the ambient tissue. Since it was not possible to remove all overgrown plates and screws without destroying their structure, the decision was made to evaluate all femora complete with the implanted osteosynthesis material. Since these small plates were relatively flexible and they were all tested under identical conditions, the obtained results were considered to be relevant. Following explantation, the subjective observations with regard to the stability were noted for each femur: stable, unstable or insecure. Next, the explantation was followed by conventional radiography. The results were rated by two observers as consolidated or not consolidated (with regard to uninterrupted callus or not).

#### Mechanical testing

The bones were stored at −20°C until mechanical testing. Mechanical testing was performed by a standardized three point bending test using a material testing device (Zwicki-line 5.0; Zwick-Roell, Ulm, Germany). The thawed bone was placed onto the device to measure the flexural stability in an anterior/posterior direction. The degree of displacement in mm at the highest pressure reached was noted. From these data the stiffness for each construct was calculated.

#### Histology

The femoral specimens were collected and decalcified in 15% formic acid at 4°C. After 14 days the screws and plates were removed. Following further processing the bones were paraffin-embedded and cut. The slides were stained with hematoxylin and eosin. Two observers identified the type of tissue adjacent to the defect area. Next, the amounts of newly formed cartilage and bone were determined using public domain ImageJ software (http://rsb.info.nih.gov/ij/). All samples were evaluated three times (same magnification for all).

### Statistical analysis

#### Sample size calculation

For determining the sample size, a mean stiffness of 15 N/mm was assumed in the control group (stabilized defects filled with β-TCP alone) with a standard deviation of 10 N/mm (obtained from preliminary results). The stiffness was expected to increase to 30 N/mm under treatment with BMP-2 and SHh. The minimum number of subjects required to detect a difference (under standard assumptions, α, 0.05; β, 0.80) was therefore eight animals in each group. To detect an even smaller difference of 13 N/mm the group number was increased to 10 animals.

#### Normality testing

The data were first analyzed for normal distribution by the D’Agostino-Pearson omnibus normality test. The majority of groups did not pass this normality test (α, 0.05). For the remaining groups, normality could not be assumed due to the small sample size.

#### Analysis of variance (ANOVA)

The data were then analyzed by non-parametric ANOVA (Kruskal-Wallis test), followed by the pairwise Dunn’s multiple comparison test.

## Results

### 

#### Surgery and mechanical testing

The surgical procedures under general anesthesia were performed without complications, although two rats died during surgery due to major bleeding. During follow-up, three more animals were sacrificed due to disabling swelling of the upper leg and nerve injury. Another animal had unclear cachexia and was sacrificed. These deceased animals were replaced by identically treated animals to maintain 10 animals in each group. The remaining animals did well and had no signs of impairment during the entire eight weeks.

**Figure 2:**
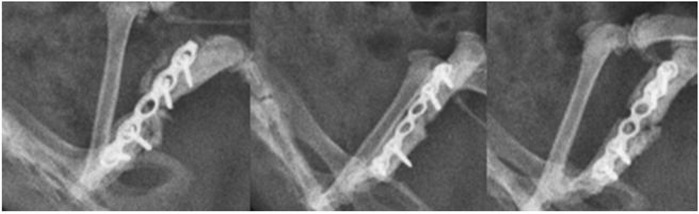
Radiological aspects of femora eight weeks after surgery. From left to right: defect filling with β-TCP/N-SHh, β-TCP/BMP-2 and β-TCP/N-SHh/BMP-2. The central femur, filled only with β-TCP/BMP-2 shows an uninterrupted callus-bridge. β-TCP, β-tricalcium phosphate; N-SHh, N-terminal sonic hedgehog protein; BMP-2, bone morphogenetic protein.

The first observation of the bones (stable, unstable or insecure) was noted when the femora were explanted. Next, the radiological evaluation was performed (Fig. 2). The femora in the N-SHh and N-SHh/BMP groups were observed to have healed poorly. The femora of the N-SHh and N-SHh/BMP groups appeared to be less stable and radiologically consolidated than their counterparts with BMP-2 and more so compared with the control with only β-TCP and no protein at all (Table II).

**Figure 3:**
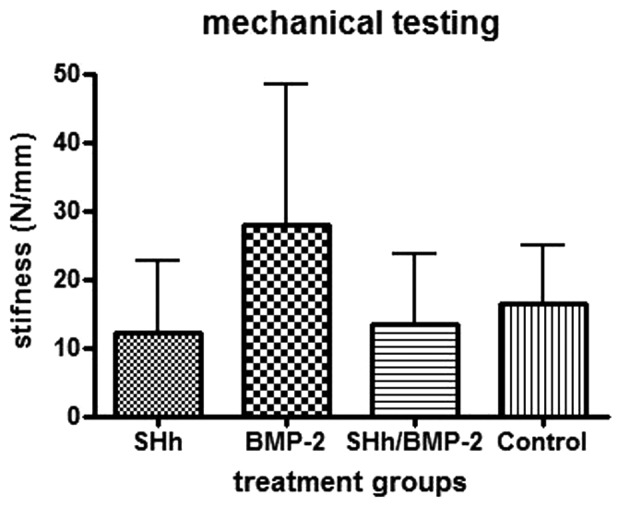
Graphical representation of the stiffness of the femora explanted from rats eight weeks after surgery (n=10 animals per group). Error bars indicate the standard deviation. Using the ANOVA Kruskal-Wallis test, no significant differences were observed (P=0.2166). N-SHh, N-terminal sonic hedgehog protein; BMP-2, bone morphogenetic protein. ANOVA, analysis of variance.

When the mechanical testing was performed, this trend was observed further. The stiffness was calculated and the femora with N-SHh and N-SHh/BMP-2 exhibited the lowest stiffness. Femora that were combined with BMP-2 were more than twice as stiff as the femora combined with N-SHh (Fig. 3).

#### Histomorphometry

The amount of newly formed cartilage and bone was calculated by ImageJ and noted as pixels per probe (whole defect area, Figs 5 and 6). With regard to the formation of new cartilage, the experimental group treated with BMP-2 and N-SHh yielded the highest amount with 410,034 pixels per sample. The animals with the N-SHh constructs had an average of 348,109 pixels per sample. The group that served as a negative control (β-TCP only) had an average of 221,426 pixels per sample. The BMP-2 group, the positive control, had the lowest average with 193,938 pixels per sample.

**Figure 4:**
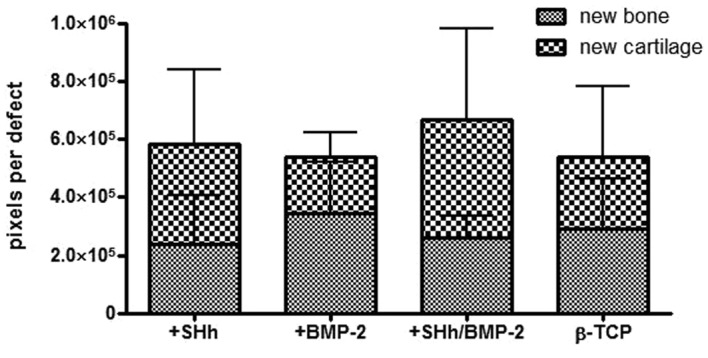
Histomorphometric distribution of newly formed cartilage and bone in the four treatment groups, measured with ImageJ. Error bars indicate the standard deviation. No mean differences for cartilage and bone were observed to be significant; ANOVA Kruskal-Wallis test; P (bone) = 0.353 and P (cartilage) = 0.0978. N-SHh, N-terminal sonic hedgehog protein; BMP-2, bone morphogenetic protein; β-TCP, β-tricalcium phosphate; ANOVA, analysis of variance.

**Figure 5:**
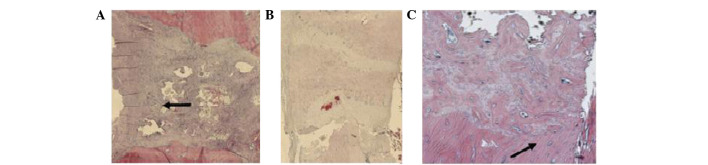
(A and B) Former defect area of an animal treated with an N-SHh/β-TCP composite. Abundant cartilage formation with little bone (arrow). (B) More bone is flanked by ossifying cartilage. (C) Defect area of a control animal (only β-TCP). Arrow indicates new bone adjacent to cartilage. N-SHh, N-terminal sonic hedgehog protein; β-TCP, β-tricalcium phosphate.

**Figure 6:**
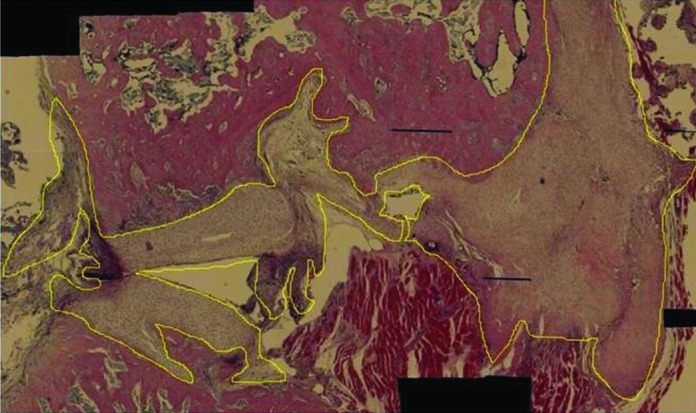
Example of analysis of cartilage formation with ImageJ (50× magnification). The yellow line encloses the measured area.

Newly formed bone was present at the highest levels in the group treated with BMP-2 (344,550 pixels per sample), followed by the control group (empty) with 291,301 pixels per sample. Less bone was observed in the group treated with BMP-2 and N-SHh (258,509) and the least amount of bone was observed in the group treated with N-SHh (231,219). The amount of newly formed bone among the groups reflected the stiffness trend observed in the mechanical testing (Fig. 4).

## Discussion

Bone defects in humans develop in various situations and frequently represent major clinical problems in bone surgery. The current treatment strategies include autologous bone transplantation, allogenous bone transplantation or, for smaller defects, filling with inorganic bone substitute materials. Autologous bone transplantation is typically restricted by donor site morbidity, limited amounts of harvested bone and longer or more extensive surgical procedures. Allogenous bone transplantation has the imminent risk of the transmission of infectious diseases and therefore requires laborious and expensive preparation procedures.

Synthetic inorganic bone substitutes, such as hydroxyapatite or TCP, are widely used and extremely biocompatible, particularly TCP which mimics the natural inorganic part of bone. The major disadvantage is the lack of osteoinductive properties. Therefore, the use of TCP is not adequate for diaphyseal or larger metaphyseal bone defects. To overcome this, TCP may be augmented by an osteoinductive protein, such as BMP-2.

In a rat model, the callus formation and bending strength of a TCP + 25 *μ*g rhBMP-2 bone graft was superior to that of an autologous bone graft. Thus, TCP/rhBMP-2 composites have the potential to become effective substitutes for autologous bone grafts (18).

To demonstrate the effect of hedgehog signaling in diaphy-seal bone defects, N-SHh impregnated β-TCP was implanted into critical-sized femoral defects of athymic rats. The defect was stabilized with the smallest available plate normally used in hand surgery. β-TCP/BMP-2 composites were used as a control. After eight weeks, the animals were sacrificed and the explanted femora were evaluated by clinical, radiological, biomechanical and histological examinations.

Notably, the N-SHh/β-TCP composites had lower stiffness than the control group consisting of β-TCP without any protein application. This effect was also observed in the N-SHh/BMP-2/β-TCP composites. The BMP-2/β-TCP composites showed the highest stiffness.

The results of the histomorphometrical analysis showed the highest amounts of cartilage in the N-SHh and N-SHh/BMP-2 groups. In this cartilage, bony islands were frequently observed, indicating a current process of endochondral ossification. The sum of the bone and cartilage was the highest in the N-SHh/BMP-2 group, which supports our initial hypothesis.

In conclusion, the addition of N-SHh at a concentration of 20 *μ*g/ml delays bone healing when added to bone substitute materials, even in combination with BMP-2. However, the amount of ossifying cartilage was the highest in the N-SHh groups, indicating a potential for robust bone healing. Further studies should incorporate longer time spans for an adequate healing period.

## Figures and Tables

**Table I:** Grouping of animals.

Group number	Number of animals	Filling of the femoral critical sized defect
1	10	β-TCP only (bone substitute material)
2	10	β-TCP + N-SHh
3	10	β-TCP + N-SHh + BMP-2
4	10	β-TCP + BMP-2

β-TCP, β-tricalcium phosphate; N-SHh, N-terminal sonic hedgehog protein; BMP-2 bone morphogenetic protein-2.

**Table II:** Values of clinical (stable), radiological (consolidated) and mechanical examination of the explanted rat femora (± indicates standard deviation).

Values	Group			
	β-TCP	β-TCP + N-SHh	β-TCP + N-SHh + BMP-2	β-TCP + BMP-2
Stable (%)	60	40	40	60
Consolidated (%)	50	10	30	50
F max (N)	47.9±28.0	33.0±23.4	38.3±27.0	59.1±7.5
Flexion (mm)	3.2±1.2	3.5 ±1.2	3.4±1.7	3.0±1.5
Stiffness (N/mm)	16.5±8.5	12.3±10.3	13.4±9.7	26.3±2.0

Between the groups and parameters, no significant differences were observed (ANOVA, Kruskal-Wallis). Not included are the six animals that died perioperatively (which were not analyzed but replaced by identically treated rats). β-TCP, β-tricalcium phosphate; N-SHh, N-terminal sonic hedgehog protein; BMP-2, bone morphogenetic protein; ANOVA, analysis of variance.
